# Arbuscular Mycorrhiza Improves Substrate Hydraulic Conductivity in the Plant Available Moisture Range Under Root Growth Exclusion

**DOI:** 10.3389/fpls.2018.00301

**Published:** 2018-03-07

**Authors:** Michael Bitterlich, Philipp Franken, Jan Graefe

**Affiliations:** ^1^Leibniz Institute of Vegetable and Ornamental Crops, Großbeeren, Germany; ^2^The Department of Plant Physiology, Humboldt University of Berlin, Berlin, Germany

**Keywords:** arbuscular mycorrhiza, unsaturated hydraulic conductivity, water retention, substrate, soil water potential

## Abstract

Arbuscular mycorrhizal fungi (AMF) proliferate in soils and are known to affect soil structure. Although their contribution to structure is extensively investigated, the consequences of those processes for soil water extractability and transport has, so far, gained surprisingly little attention. Therefore we asked, whether AMF can affect water retention and unsaturated hydraulic conductivity under exclusion of root ingrowth, in order to minimize plant driven effects. We carried out experiments with tomato inoculated with *Rhizoglomus irregulare* in a soil substrate with sand and vermiculite that created variation in colonization by mixed pots with wild type (WT) plants and mycorrhiza resistant (RMC) mutants. Sampling cores were introduced and used to assess substrate moisture retention dynamics and modeling of substrate water retention and hydraulic conductivity. AMF reduced the saturated water content and total porosity, but maintained air filled porosity in soil spheres that excluded root ingrowth. The water content between field capacity and the permanent wilting point (6–1500 kPa) was only reduced in mycorrhizal substrates that contained at least one RMC mutant. Plant available water contents correlated positively with soil protein contents. Soil protein contents were highest in pots that possessed the strongest hyphal colonization, but not significantly affected. Substrate conductivity increased up to 50% in colonized substrates in the physiologically important water potential range between 6 and 10 kPa. The improvements in hydraulic conductivity are restricted to substrates where at least one WT plant was available for the fungus, indicating a necessity of a functional symbiosis for this effect. We conclude that functional mycorrhiza alleviates the resistance to water movement through the substrate in substrate areas outside of the root zone.

## Introduction

Biostimulants in agri- and horticulture are defined as substances or microorganisms applied to plants in minute quantities aiming to improve crop quality traits, stress tolerance and nutrient efficiency, without being mineral nutrients, soil improvers or pesticides, which are applied in high quantities (du Jardin, 2015). Biostimulants include humic acids, protein hydrolysates, seaweed extracts, biopolymers (Colla et al., 2015; du Jardin, 2015), beneficial microbes such as plant growth promoting rhizobacteria (Ruzzi and Aroca, 2015) and arbuscular mycorrhizal fungi (AMF) (Rouphael et al., 2015).

Arbuscular mycorrhizal fungi proliferate in soils beyond the root zone. Thus, extraradical hyphae constitute a network that enhances the soil volume connected to the plant (Smith and Read, 2008). Along with the well investigated phosphorus delivery of AMF to plants from beyond root zone areas (Smith and Read, 2008), AMF influence soil properties. Soil structure, the three-dimensional particle arrangement of organic/mineral units, i.e., aggregates, and pore space is of substantial importance for nutrient, gas and water fluxes in soils (Wu et al., 2014). AMF presence, activity and turnover affects soil structure (Augé et al., 2001; Piotrowski et al., 2004; Rillig and Mummey, 2006; Leifheit et al., 2014). Similar to roots (Bodner et al., 2014), AMF contribute substantially to the hierarchical formation of aggregates by merging smaller aggregates of the sediment load into larger macroaggregates (Tisdall and Oades, 1982; Miller and Jastrow, 2000; Six et al., 2004; Rillig and Mummey, 2006). Increased abundance and stabilization of aggregates by AMF hyphae are frequently observed, well documented and related to their so called ‘sticky-string bag’ function (Miller and Jastrow, 2000). Those hyphae entangle and enmesh particles due to polysaccharides and other ‘sticky’ substances on their surface to which solid particles adhere. Hence, hyphae stabilize particle-particle contact and form aggregates (Degens et al., 1996) or maintain particle connectivity by bridging pore spaces (Miller and Jastrow, 2000; Rillig and Mummey, 2006). In addition, turnover of mycelia releases organic substances into soils (Driver et al., 2005; Rillig and Mummey, 2006). Glomalin, a proteinaceous substance produced by AMF, is mainly released during hyphal turnover (Driver et al., 2005). As a part of the slow turnover carbon pool, glomalin accumulates in many soils and is often positively correlated to aggregate stability (Wright and Upadhyaya, 1998), because it sticks particles together and/or hinders disintegration of those organic/mineral units by water infiltration. AMF may also produce hydrophobins, proteins of filamentous fungi that lower the surface tension of water and enables hyphae to break through a water film (Rillig, 2005). Hydrophobins may contribute to observations that AMF can increase soil water repellency (Rillig et al., 2010). Thus, AMF can influence the physical properties of soils in various direct and also indirect ways, i.e., changes to microbial community, host morphology and activity, which may act in concert, in antagonistic or synergistic ways (Rillig and Mummey, 2006). The structure-related changes in physical soil properties and surfactant effects of released biochemicals determine water and solute mobility, as well as transport capacity of the growth soil or substrate. Hence, soils colonized by AMF may possess different constraints to solute and water transport. Currently, effects of AMF on soil structure may be consensus, but the relevance of those effects to plants remains less clear (Querejeta, 2017).

Water and solute transport occurs through the pore space. The hydraulic properties of the pore space are related to primary texture, i.e., particle size distribution, and secondary structure (De Gryze et al., 2006; Querejeta, 2017). Thus, by changing the structure of a soil, AMF can influence the soil’s hydraulic properties (Augé et al., 2001; Augé, 2004). Hitherto, comparative quantification of the hydraulic properties of soils with and without mycorrhiza has gained surprisingly little attention (Querejeta, 2017), even though they limit the mass flow of water and solutes to roots and the ability of plants to extract water. Water retention and hydraulic conductivity are quantitative measures for water capacity and mobility, respectively, but water retention has been rarely quantified for mycorrhizal substrates (Augé et al., 2001; Bearden, 2001; Augé, 2004; Daynes et al., 2013) and, to the best of our knowledge, unsaturated hydraulic conductivity of colonized substrates is not yet reported at all. In substrates that contain roots as well as AMF, water retention relations can shift compared to non-colonized substrates (Augé et al., 2001; Bearden, 2001; Daynes et al., 2013). The pioneering study of Augé et al. (2001) revealed that water contents of equally rooted mixtures of sandy soil corresponded to different water potentials. The soil water potential equals the energy plant roots require to extract water from the soil, achieved by root osmotic adjustments. Plant physiological responses to AMF colonization can be related to direct mycorrhizal effects on root hydraulic conductivity and regulation of root aquaporin expression (Porcel et al., 2006; Aroca et al., 2007; Bárzana et al., 2012). Additionally, they could be related to shifts in soil water retention and conductivity due to AMF colonization. Indeed, many studies measuring the physiological response of shoots to soil moisture found that, in order to trigger a comparable response to non-colonized soils, AMF soils had to be drier (reviewed in: Augé, 2001). In another study, mycorrhiza-resistant bean mutants showed improved stomatal conductance when grown in a mycorrhizal soil (Augé, 2004). In order to avoid or delay wilting, plants adjust stomatal aperture to the soil water status (Tardieu and Simonneau, 1998). Hence, the observations by Augé (2004) point to a soil-originated effect.

Compartments that exclude root and allow fungal ingrowth have not yet been used to assess water retention and conductivity. Yet, they could give a better idea of the impact of extraradical hyphae in non-rooted substrates on plant water supply. On a small scale, non-rooted areas like these probably occur in every mycorrhizal scenario. Root water uptake can form water depletion gradients between rooted and non-rooted areas. Measuring the hydraulic properties inside the root-free compartments could serve as a surrogate for water extractability and water transport away from those areas. We investigated whether AMF can change water retention and hydraulic conductivity in root-free substrates. To test this, we designed an experiment using the root-free compartments and mixed cultures of host and non-host tomato genotypes in order to create a variation of substrate colonization, similar to previous approaches (Neumann and George, 2005a; Hallett et al., 2009). We chose a substrate mix that allows for growing plants hydroponically. It resembles two or three component substrate mixtures, comprising sand and vermiculite that are frequently encountered in fundamental mycorrhizal research (e.g., Azcón, 1989; El-Atrach et al., 1989; Kim et al., 1997; Boldt et al., 2011; Porcel et al., 2015, 2016; Rasmussen et al., 2016; Ruiz-Lozano et al., 2016) and, for inoculum production (Silva et al., 2007). We used a hydroponic system to minimize the effect of reduced accessible substrate volume in non-mycorrhizal (NM) pots. We frequently replaced nutrients that were taken up with low quantities of readily available nutrients. This way, the additional volume accessible by hyphal ingrowth in mycorrhizal pots only has a marginal impact on plant nutrition and, thus, reduces nutrient-related growth responses. Moreover, AMF effects on soil hydraulic properties are expectably most pronounced in coarsely textured environments (Leifheit et al., 2014; Querejeta, 2017). We used tomato as a host, a rather non-responsive plant to AMF in terms of growth (e.g., Smith et al., 2004), to minimize differences in root-compartment contact and irrigated daily to minimize pronounced dry-wet cycles in those compartments.

## Materials and Methods

### Experimental Design, Plant Growth, and Mycorrhizal Development

We planted two *Solanum lycopersicum* cv. 76R wild type (WT) plants and the mutant resistant to mycorrhizal colonization (RMC) (Barker et al., 1998) in 7.5 L pots in intra- (WT/WT; RMC/RMC) and inter-genotypic (WT/RMC) combination and inoculated half of the pots with a commercial inoculum containing *Rhizoglomus irregulare* (INOQ GmbH, Schnega, Germany). Four replicates were randomly set up in the greenhouse, grown for 8 weeks and irrigated daily with 600 mL nutrient solution (De Kreij et al., 1997); N: 10.32 mM; P: 0.07 mM; K: 5.5 mM; Mg: 1.2 mM; S: 1.65 mM; Ca: 2.75 mM; Fe: 0.02 mM; pH: 6.2; EC: 1.6 mS) containing 10% of standard phosphate to guarantee good fungal colonization. Additional 400–600 mL of deionized water were added daily, so that total irrigation maintained 13–16% volumetric water content (10–30 kPa). The pots contained a sterilized mixture (2:2:1; v/v/v) of fine sand (0.2–1 mm; Euroquarz, Ottendorf-Okrilla, Germany), vermiculite (Agra-Vermiculite, Rhenen, Netherlands) and 2 mm sieved local sandy soil (per 100 g soil: 5.3 mg P; 5.4 mg K; 1.9 mg Mg; 1.1 mg N; organic matter 1.3% DW; pH 6.1; 82% sand; 14% silt; 4% clay). The reproducible substrate allowed hydroponic fertilization to better control nutrient availability. The soil added served as a source for aggregation nuclei from some smaller sediment and homogeneously distributed organic matter. Two 250 mL compartments were introduced into the pots with a 30 μm nylon mesh that excludes root ingrowth. One was used to extract extraradical mycelia [containing a mixture of glass beads and soil as in Neumann and George (2005b)] to verify extraradical spreading and the other containing the potting mixture to assess moisture retention properties.

The pots were inoculated with 0.5 L of the *R. irregulare* inoculum. For the non-mycorrhizal treatment the inoculum was filtered with deionized water and autoclaved for 15 min at 121°C. The filtrate and the sterilized inoculum were added to the pots in equal amounts (0.5 L). The compartments were not inoculated.

Bradford-reactive soil protein content (BRSP) was quantified (triplicates; 2 g subsamples of air dried substrates) after Wright and Upadhyaya (1996) as a measure for glomalin, an important compound for aggregate stability with surfactant properties. Trypan blue staining was carried out after Koske and Gemma (1989) for intrardical staining of AMF and intraradical colonization was quantified on 100 root pieces with the grid line intersection method after Giovannetti and Mosse (1980).

For shoot phosphorus (P), 250 mg of pulverized dry material was oxygenized for 20 min with 5 mL HNO_3_ (65%) and 3 mL H_2_O_2_ (30%). After 1 h of microwave decomposition and filtration (MN 615, Macherey-Nagel, Germany) P concentration of filtrates were analyzed with an EPOS analyser (ascorbic-acid method, EPOS 5060-55, Eppendorf, Germany).

### Assessment of Substrate Hydraulic Properties

The bulk density in compartments was 1.2 g cm^-3^, calculated as the oven-dried (24 h; 105°C) weight per unit volume. Air filled porosity is determined as the difference of dry porosity and the saturated water content (Θ_SAT_). Before planting, standard soil sampling cores (*V* = 250 mL, *h* = 5 cm) were introduced into pots (*n* = 4 per treatment and planting combination) in a way that the cylinder diameter covered the central section of the substrate filling level and the depth of the cylinder covered the radius from the center to the rim of the pot.

After harvesting, the simplified evaporation method (Schindler, 1980) was applied on the sampling cores introduced into the substrate fraction that excluded root proliferation. The principle of the simplified evaporation method is a continuous drying of a soil or substrate sample under controlled laboratory conditions. This measurement was carried out with a commercial HYPROP system (UMS GmbH, München, Germany) using standard sampling cores (*V* = 250 mL, *h* = 5 cm). For the measurement in the HYPROP system, the soil cores were weighed, water saturated overnight and two tensiometers were introduced into the substrate using carefully prepared holes. The water tension of both tensiometers (h_1_, h_2_, and hPa) was logged every 10 min during evaporation and the substrate samples were weighed at least two times a day. Using the mean soil water potential (Ψ_S_ and hPa) over both tensiometers and the weight loss from evaporation, a retention function of the mean volumetric water content [Θ(Ψ_S_)] of the prepared soil sample can be derived (Peters and Durner, 2008). The measurement lasted until the know air entry point of the tensiometers ceramic (Al_2_ O_3_ and 8800 hPa) was passed, when tension drops off to 0 hPa. This specific can be used to extent the typical measurement range of tensiometers from 1000 to 8800 hPa (Peters and Durner, 2008). Afterward, samples were dried (105°C, 24 h) and weighed again to determine substrate dry mass.

Assuming half of the water flow for evaporation deriving from the upper cylinder height and a linear gradient of volumetric water content (Θ) from bottom to top, a function for the hydraulic conductivity *K*(*h*) can be estimated as:

(1) $$\begin{array}{c} K(h_{i})=\;\frac{0.5q}{\frac{\Delta h}{z_{1}-z_{2}}-1} \end{array}$$

where *q* is the water flow, Δh is the mean tension difference of the two tensiometers and *z*_i_ are the depths of the tensiometers (Peters and Durner, 2008).

Although made for soils, water retention models can also be used for substrate mixes (Fonteno, 1992). Thus, several water retention models were tested. In order to evaluate the functional relationship between Θ and Ψ_S_, a bimodal model for water retention (Durner, 1994) was fitted with the HYPROP-DES software (UMS GmbH, München, Germany). The adopted model allows for a mixture of two pore size distributions (*n*_1_, *n*_2_) (Eq. 2). This model choice was based on the Akaike Information Criteria (AICc) for finite sample sizes (Akaike, 1974), which penalizes model complexity, so candidate models with minimum AICc are preferred.

(2) $$\begin{array}{c} \;S_{e}(h)=\sum_{i=1}^{2}\omega_{i}{\left(\frac{1}{1+{\left(\alpha_{i}\left|\psi_{S}\right|\right)}^{n_{i}}}\right)}^{1-\frac{1}{n_{i}}} \end{array}$$

*S*_e_ is the effective saturation defined as *S*_e_ = (Θ - Θ_r_)/(Θ_SAT_ - Θ_r_) (Mualem, 1976) with Θ_r_ and Θ_SAT_ as the saturated and residual volumetric water content, respectively. ω_i_ is a weighting factor for the specific mixture component, *n*_i_ is the pore size distribution parameter and α_i_ is the reciprocal potential at the air entry water tension of the substrate sample. The input is the geometric mean of tensions of both tensiometers (Ψ_S_).

The retention function was coupled to a model of for hydraulic conductivities (*K*) in unsaturated porous media (Mualem, 1976):

(3) $$\begin{array}{c} K\left(h\right)=\;K_{s}S_{e}^{\tau }{\left(\frac{\int_{0}^{S_{e}}h^{-1}\;dS_{e}\left(h\right)}{\int_{0}^{1}h^{-1}dS_{e}\left(h\right)}\right)}^{2} \end{array}$$

where *K*_S_ and τ are the saturated conductivity and a pore tortuosity parameter, respectively. Overall nine parameters were identified simultaneously from combined retention and conductivity data: Θ_S_, Θ_r_, *w*_2_, α_1_, α_2_, *n*_1_, *n*_2_, *K*_S_, and τ. Parameter estimation was carried out as described in Peters and Durner (2008).

Statistical analysis (α = 0.05; normal distribution, homogeneity of variances, ANOVA, *t*-test and regressions) and figures were done with STATISTICA 12 Software (StatSoft, Tulsa, OK, United States).

## Results

As anticipated, plant growth and P content were not changed by inoculation with *R. irregulare* (**Table 1**). Substrate characteristics in the root-free compartments were largely unaffected by plant combinations. Porosity and the saturated water content (Θ_SAT_) were reduced in mycorrhizal substrates. Unaltered air filled porosity indicates that *R. irregulare* reduced the effective wettable pore space. Bradford-reactive soil protein contents (BRSP) neither changed upon plant genotype combination nor upon inoculation. Changes in Θ_SAT_ were not related to BRSP contents (*R*^2^ = 0.10, *P* > 0.05).

**Table 1:** Substrate and plant parameters of non-mycorrhizal (NM) pots and pots inoculated with *Rhizoglomus irregulare* (AM) for the intra- and inter-genotypic plant combinations containing either two wild type tomato plants (WT/WT), two resistant mutants (RMC/RMC) or one WT and one resistant mutant (WT/RMC).

Variable	Inoculation	Pot combination			ANOVA		
		WT/WT	WT/RMC	RMC/RMC	Pot	Inoculation	H × I
**Substrate**							
Total dry porosity [cm^3^ cm^-3^]	NM	0.56 ± 0.02	0.54 ± 0.01	0.56 ± 0.01	0.516	**0.026**	0.727
	AM	0.53 ± 0.01	0.53 ± 0.01	0.53 ± 0.01			
Θ_SAT_ [cm^3^ cm^-3^]	NM	0.50 ± 0.01	0.49 ± 0.01	0.50 ± 0.01	0.652	**0.031**	0.885
	AM	0.47 ± 0.01	0.48 ± 0.01	0.49 ± 0.01			
Air filled porosity [cm^3^ cm^-3^]	NM	0.06 ± 0.02	0.05 ± 0.01	0.06 ± 0.01	0.784	0.698	0.906
	AM	0.06 ± 0.02	0.05 ± 0.01	0.05 ± 0.01			
*K*_SAT_ [log10 cm d^-1^]	NM	2.00 ± 0.06	2.01 ± 0.19	2.14 ± 0.19	0.414	0.856	0.443
	AM	1.91 ± 0.09	2.23 ± 0.12	2.07 ± 0.08			
BRSP [mg cm^-3^]	NM	0.38 ± 0.08	0.42 ± 0.06	0.43 ± 0.05	0.771	0.258	0.204
	AM	0.47 ± 0.09	0.46 ± 0.06	0.40 ± 0.04			
**Plant**							
Plant dry weight [g]	NM	148 ± 3.94	149 ± 8.35	159 ± 6.13	0.913	0.822	0.455
	AM	151 ± 10.4	154 ± 4.88	147 ± 8.09			
Root dry weight [g]	NM	37.5 ± 3.22	36.4 ± 4.41	45.9 ± 6.22	0.911	0.837	0.204
	AM	40.1 ± 5.15	42.0 ± 4.38	35.5 ± 3.30			
Shoot phosphorus [% DW]	NM	0.28 ± 0.01	0.27 ± 0.02	0.26 ± 0.01	0.619	0.206	0.849
	AM	0.25 ± 0.02	0.26 ± 0.01	0.25 ± 0.01			

***Θ**_*SAT*_ and K_*SAT*_ denote the saturated water content and conductivity, respectively. Means (±SE) and significant P-values are highlighted in bold (two way ANOVA, α = 0.05, n = 4).*

**FIGURE 1:**
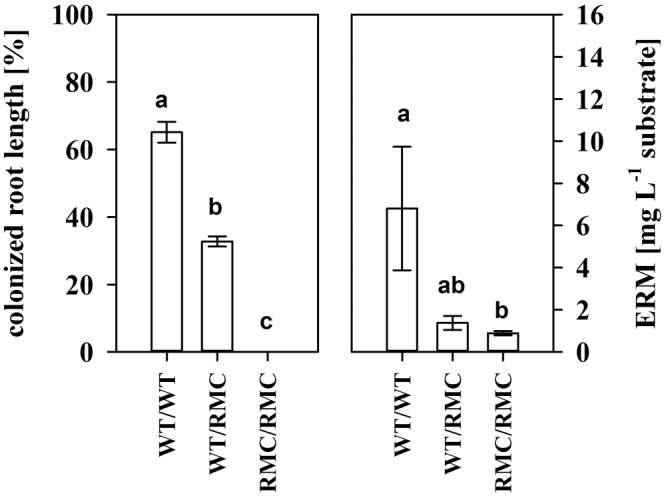
Colonized root length (left) and the extracted extraradical mycelia (ERM) dry matter of pots inoculated with *Rhizoglomus irregulare* for the intra- and inter-genotypic plant combinations containing either two wild type tomato plants (WT/WT), two resistant mutants (RMC/RMC) or one WT and one resistant mutant (WT/RMC). No colonization was observed in non-mycorrhizal pots. Means (±SE) are shown and values with the same letter are not significantly different (one way ANOVA, Tukey HSD, α = 0.05, *n* = 4).

In pots that contained two RMC mutants, we only observed surface colonization of roots (**Figure 1**). Non-mycorrhizal (NM) roots were free of mycorrhiza. Colonization of the bulk root system was reduced in the inter-genotype planting combination compared to roots from pots with two WT plants. Although only surface colonization was detected, we found a small amount of hyphae also in root-free compartments from pots with two RMC mutants. Since we did not find any hyphae in NM compartments and the compartments were not inoculated, those hyphae have to originate from growing fungi.

The water retention and hydraulic conductivity analyses were carried out in a pot specific manner because, in a few instances, variation in individual assessments caused inflections in treatment-wise model fits that diverged from individual curvatures.

**FIGURE 2:**
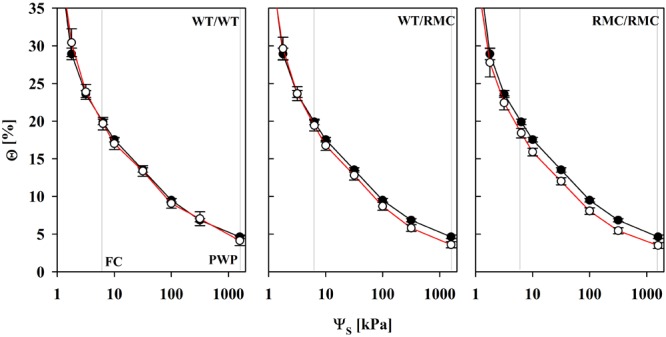
Water retention curves of non-mycorrhizal (black line) pots and of pots inoculated with *R. irregulare* (red line) for intra- (WT/WT; RMC/RMC) and inter-genotypic (WT/RMC) plant combinations from field capacity (FC) to the permanent wilting point (PWP). Θ and Ψ_S_ denote the volumetric water content and the substrate water potential, respectively. Means (*n* = 4; ± SE) are shown, deriving from individual pot specific fits. There was a significant mycorrhizal effect at Θ_100kPa_ (*P* = 0.016) and Θ_1500kPa_ (*P* = 0.015). Marginal significances were observed for Θ_10kPa_ (*P* = 0.054) and Θ_300kPa_ (*P* = 0.096). Significant differences between planting combinations or factor interaction were not detected (two way ANOVA, α = 0.05, *n* = 4).

**FIGURE 3:**
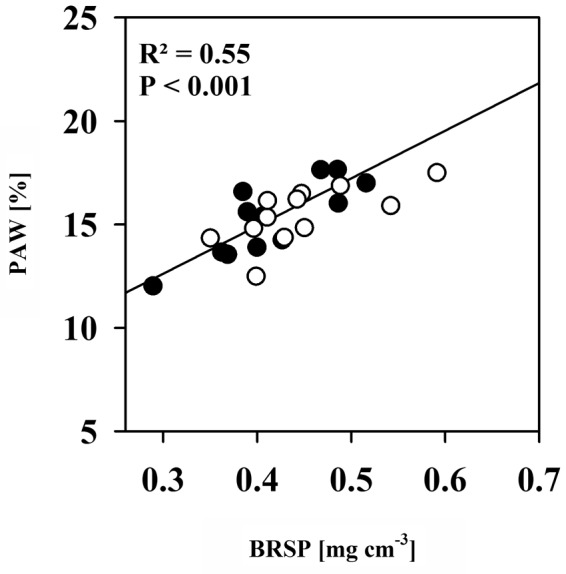
The relationship between plant available water contents (PAW) and Bradford-reactive soil protein contents (BRSP) for non-mycorrhizal (black dots) pots and of pots inoculated with *R. irregulare* (white dots) for all intra- and inter-genotypic plant combinations combined per inoculation treatment. Significant differences between planting combinations, mycorrhizal inoculation or factor interaction were not detected (two way ANOVA, α = 0.05, *n* = 4).

The plant relevant range in most scenarios complies with water potentials from field capacity (FC; Ψ_S_ = 6 kPa) to the permanent wilting point (PWP; Ψ_S_ = 1500 kPa). Within this range, the water content of colonized substrates was gradually lower with every RMC plant added to the pots, indicating that those substrates can be more thoroughly depleted of water (**Figure 2**). Θ at FC was equal in NM and AMF substrates, but was lower at the PWP. This difference was most pronounced in RMC/RMC pots. However, the total plant available water content between FC and PWP was not different in colonized substrates and was equally related to the amount of BRSP in NM and AMF substrates (**Figure 3**). The mycorrhizal effect that relates to the shift in water retention was observed between Ψ_S_ = 3 and 6 kPa. Here, the slope of the curve was steeper in AMF substrates (*P* = 0.042), which was not related to the planting combination (*P* = 0.458). Exemplarily, an additional depletion of approximately 2.4% water content was required in colonized WT/WT pots to reduce Ψ_S_ by 3 kPa, whereas less than 1% of additional reduction in Θ was required in the other planting combinations.

**FIGURE 4:**
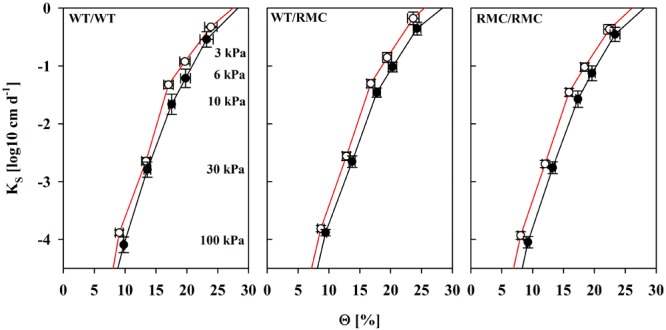
Substrate hydraulic conductivity (*K*) as a function of the volumetric water content (Θ) of non-mycorrhizal (black line) pots and of pots inoculated with *R. irregulare* (red line) for intra- (WT/WT; RMC/RMC) and inter-genotypic (WT/RMC) plant combinations. Means (*n* = 4; ±SE) are shown, deriving from individual pot specific fits. There was a significant mycorrhizal effect at *K*_6kPa_ (*P* = 0.040) and Θ_10kPa_ (*P* = 0.034). Marginal significances were observed for Θ_3kPa_ (*P* = 0.072) and Θ_100kPa_ (*P* = 0.067). Significant differences between planting combinations or factor interaction were not detected (two way ANOVA, α = 0.05, *n* = 4).

**FIGURE 5:**
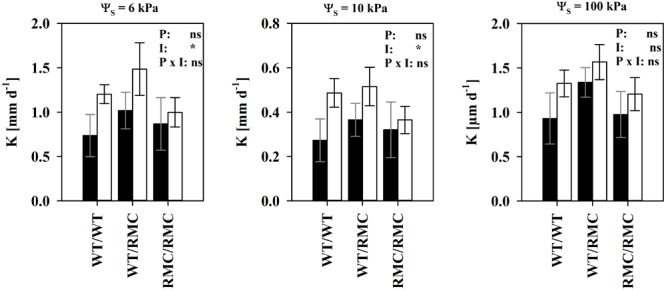
Substrate hydraulic conductivity (*K*) at three different levels of substrate water potential (pF) of non-mycorrhizal (black columns) pots and of pots inoculated with *R. irregulare* (white columns) for the intra- and inter-genotypic plant combinations containing either two wild type tomato plants (WT/WT), two resistant mutants (RMC/RMC) or one WT and one resistant mutant (WT/RMC). Means (*n* = 4; ±SE) are shown, deriving from individual pot specific fits. Asterisks denote significant differences between non-mycorrhizal and mycorrhizal pots. Significant differences between planting combinations or factor interaction were not detected (two way ANOVA, α = 0.05, ^ns^*P* > 0.05, ^∗^*P* < 0.05).

Considering equal suction and water content, hydraulic conductivity determines substrate the water flow capacity. We found unsaturated hydraulic conductivity (*K*) improved in colonized substrates between Ψ_S_ = 6 kPa and Ψ_S_ = 10 kPa (**Figure 4**). The effect was most pronounced in WT/WT pots and gradually decreased with the replacement of one or two WT plants with RMC mutants. This range is the most relevant for plant water uptake in most scenarios, because water is held against gravitation and easily extractable in high quantities. This effect was truly a mycorrhizal one. Although variability in *K* was also partially explained by BRSP [*K*(Ψ_S_ = 6 kPa): *R*^2^ = 0.31; *P* < 0.05; *K*(Ψ_S_ = 10 kPa): *R*^2^ = 0.41; *P* < 0.05; *N* = 24], the mycorrhizal improvements in *K* were still significant [*K*(6 kPa): *P* = 0.028; *K*(10 kPa): *P* = 0.041], when BRSP was used as the continuous predictor to account for BRSP variability. To some degree, *K* in mycorrhizal substrates depends on more than the protein content and, interestingly, this positive effect seemed to require a functional symbiosis. Expressed on an absolute basis, true enhancements were only observed in pots where at least one WT was growing, but totally absent in the RMC/RMC pots (**Figure 5**). At higher water potentials values those mycorrhizal influences were less pronounced.

## Discussion

We succeeded to obtain plants of similar size with equal nutritional status, which was important to minimize the plant’s influence on root-free compartments. Non-mycorrhizal treatments for every pot combination were required, because the substrate without roots is nevertheless in physical contact with the surrounding rooted substrate. It is, thus, not entirely uncoupled from plant activity, i.e., via substrate mass flow driven by transpiration, as indicated in a similar approach with the same genotypes (Hallett et al., 2009). However, we did not detect any changes in substrate-related traits upon the planting combination. Hence, we assume that the impact of plants on root-free compartments was equal in all combinations.

We found also mycelia in pots where only resistant mutants grew. This is possible, because spore germination of AMF mainly depends on soil moisture and temperature (Daniels and Trappe, 1980) and pre-symbiotic hyphae can proliferate rather undirected over distances of almost 1 cm (Powell, 1976). That would cover half of the sampling core depth, when colonized from both entry sides. We assume this also happened in compartments subjected to water retention assessments.

### Mechanisms Affecting Substrate Hydraulic Characteristics

When AMF penetrate soils or substrates, they grow in pores and on the surface of particles. In this way, they influence the size of the pore space and change its physico-chemical properties by releasing biochemicals, inducing particle redistribution and altering the degree of particle surface coverage with organic material. Penetrating AMF can induce aggregation of particles, i.e., affect soil structure, by entangling and enmeshing solid particles and by releasing polysaccharides and other ‘sticky’ substances to which particles adhere. They furthermore change surface wettability, because organic materials possess other wetting properties than mineral particles. Aggregation affects pore size distribution (Daynes et al., 2013), pore geometry and, surfactant effects influence the wettable pore space and water extractability. In the bulk pore space (quantified as porosity from the ratio of dry bulk density and particle density), water and solute transport occurs. Its water storage and transport capacity is measured by water retention and hydraulic conductivity characteristics that depend on those structural and surfactant effects.

Porosity increases when organic matter is removed by combustion (McCarthy et al., 2008). AMF are part of the organic matter pool. Moreover, AMF can increase water repellency (Rillig et al., 2010) and may therefore be responsible for the slight reduction in the total wettable pore space, i.e., the saturated water content (Θ_SAT_). One could argue that the reduction in Θ_SAT_ upon substrate colonization were merely due to the volume of fungal biomass present. However, to achieve this, more than 22000 spores (with a radius of 60 μm) or 63 km of hyphae (with a diameter of 10 μm) per cm^3^ of substrate would be required. These are unlikely dimensions. Still, there could be a small impact because spores and hyphae will at least be partially attached to the solid matrix, potentially blocking small pore necks, and could impede access to larger continuous pore spaces (Rillig et al., 2007). This would require that those pores had been air filled, when samples were harvested. Factors determining water repellency change the liquid-solid phase contact angle (Letey et al., 1962; Hallett, 2008) and potentially reduce the effective wettable pore space. This may have been induced by AMF. Indeed, water contents near saturation drop with repellency (Hallett, 2008). This is consistent with our observed restoration of air filled porosity and could also have hindered infiltration of pores during saturation, which were only partially clogged by a hydrophobic fungal structure. Water repellency effects are especially pronounced in coarsely textured substrates, where the main driver for repellency, i.e., organic matter including AMF, covers a relatively high proportion of solid surface (Doerr et al., 2000; Hallett, 2008). In principle, the substrate we used falls into this category as it was dominated by coarse sand and large vermiculite particles (<200 μm). Still, the differences, although significant, have been small and possibly could have been abolished upon longer duration of water contact during saturation (Doerr et al., 2000).

The shape of the water retention curves of soils are determined by the primary, i.e., particle size distribution, and the secondary structure of the pore system, the latter being shaped by aggregation and formation of microchannels (Durner, 1992). Depending on size and genetic development of aggregates, shifts of water retention due to the structural pore system are located between Ψ_S_ = 0.3 and 10 kPa (Durner, 1992). AMF influence the mean aggregate diameter (Daynes et al., 2013). The size and amount of aggregates alters the proportions of inter-aggregate and enclosed intra-aggregate pores of the total pore space (Regelink et al., 2015). Those discrete structural changes to pore size distribution in the bulk pore system peak in narrow to moderate ranges (Durner, 1992) and require a detailed look. Indeed, a steeper slope of [Θ(Ψ_S_)] within the expected range (here within Ψ_S_ = 3–6 kPa) was observed in colonized substrates. This is consistent with other reports on a sandy soil mixture (Augé et al., 2001), a vertisol (Bearden, 2001) and a substrate deriving from coarse spoil (Daynes et al., 2013) under root ingrowth. We show here for the first time that this pattern also appears under root exclusion. Because this was most pronounced in WT/WT pots in the current experiment, changes in the structural pore system seemed to be related to the viability or activity of the symbiosis.

Soil protein contents, here quantified as BRSP, are often positively correlated with aggregation and subsequently with AMF colonization (e.g., Rillig et al., 2002; Wu et al., 2012). Within aggregates, pores of particular sizes exist that hold water against gravitation and are emptied within the plant-available moisture range (Hallett et al., 2009; Regelink et al., 2015). If AMF promotes aggregation, increases in water contents between FC and PWP would hence be expected. Consistently, BRSP contents were positively correlated with plant available water contents in the moisture range between FC and PWP (**Figure 3**). However, neither BRSP contents nor plant available water contents were influenced by AMF. Hence, there was no effect of AMF on total plant available water. We only found the tendency that BRSP improves upon substrate colonization. In general, however, levels were very variable. BRSP accumulates in soils because of its low turnover rate and is mainly released by AMF during hyphae turnover (Driver et al., 2005). We have provided a substantial amount of BRSP with the soil (as indicated by contents from NM pots). This was more important for water holding capacity than the possibly minute quantities added by AMF. Due to the rather short duration of the experiment, BRSP release from hyphae turnover was probably low.

Interestingly, in substrates from WT/RMC and RMC/RMC pots, lower water contents were observed in the plant-available moisture range, but not in WT/WT pots. In pots containing a viable symbiosis, the water contents between FC and PWP were conserved, which may indicate structural stabilization under the absence of roots. The reduction of water contents between FC and PWP in WT/RMC and RMC/RMC pots in comparison to their NM counterparts indicates a destabilization of structure and losses of structural pore volume. We can only speculate about underlying mechanisms. One scenario could be that this observation is related to the absence of access to plant derived carbon in a-symbiotic fungi. A-symbiotic AMF will lack plant carbon to produce compounds required to adhere to particles or cover surfaces, but may invest resources from spores in proliferation toward susceptible hosts. It follows that effects of stickiness may be outweighed by penetration. Macroaggregates formed by fungi are transient and underlie turnover (Six et al., 2004). We may have provided such aggregates by the addition of soil (like BRSP), which could have been partially disintegrated by penetration of less ‘sticky’ a-symbiotic fungi. This would increase the access to previously enclosed intra-aggregate pores and water may become accessible upon disintegration. Another possibility coming from the same effect is that a-symbiotic hyphae could induce less pore enclosure when growing inside aggregates or on the surface of particles. Although, the scenario is speculation and requires further research, it would also explain the maintenance of water retention in NM substrates that not possessed penetrating organisms and would not require observable changes in protein contents.

On coarsely textured substrates like sandy soils, AMF effects are mainly related to the sticky-string bag function (Miller and Jastrow, 2000) inducing entanglement and enmeshment processes with hyphae bridging large pore spaces between particles (Clough and Sutton, 1978; Forster, 1979). This is a realistic scenario also for our substrate and may be explanatory for the stimulation of unsaturated hydraulic conductivity between Ψ_S_ = 6 and 10 kPa. We show that here for the first time. Unsaturated hydraulic conductivity is mainly determined by the few largest water filled pores and in addition, depends on pore tortuosity and pore connectivity (Durner, 1994). Unsaturated conductivity can change although water retention remains the same (Durner, 1992), which is exactly what we observed in WT/WT pots. The shape and organization of voids in aggregated soils differs from unstructured porous systems. As an example, a net of planar voids will show a quite different hydrological behavior than a pore space between spherical aggregates, although both may possess similar retention characteristics (Durner, 1992). Under the present conditions around field capacity, large pores are already air filled and hyphae may constitute a connection for water and/or a less tortuous pathway for water. A less tortuous water pathway would also appear, when hyphae have a smoothing effect on particle profiles. Like other microbes, AMF may also produce compounds that are hydrophobic when dry, but strongly hydrophilic when wet (Hallett, 2008). This could increase conductivity from those water filled pores. Due to the dimension of hyphae, it is unlikely that just intrahyphal water flow explains this observed promotion, which was as large as 50% in this study. This is further supported by the fact that substrates from pots with two resistant mutants completely lack that promotion. This is interesting and worth studying in detail in the future. The absence of stimulation of *K* in substrates with a-symbiotic hyphae may point to the necessity of a functional symbiosis that has access to plant derived carbon as a source for production of exudates and sticky substances.

Further studies can elucidate the proposed mechanisms of this discussion, e.g., by applying imaging technologies on the microscale. The superior aim of this study was to quantify AMF impacts on substrate hydraulic properties under conditions similar to those frequently encountered in mycorrhizal research that target physiological host responses. Stabilization of water retention and improvements of unsaturated hydraulic conductivity outside root zone areas were apparently most pronounced in systems with the most viable symbiosis. It would be fascinating to combine outcomes of soil hydraulic properties with molecular techniques, elucidating the expression of genes required to produce those AMF compounds that influence the physical properties of soils in viable symbioses. In particular it would be intriguing to investigate whether the expression of genes encoding for production of glycoproteins and hydrophobins requires a symbiotic association with plants. More importantly, we did not find a scenario, where substrate hydraulic properties of colonized pots equaled that of non-colonized ones. This may have important consequences for plant reactions, which will, however, differ with the setting.

### Relevance for Plants

It is consensus that plants grow differently on different soils under otherwise equal conditions. This may also apply for AMF plants in the same soil or substrate with different properties. Our findings underpin the extension of the ambit of plants by AMF hyphae, not only for P acquisition, but also for their hydraulic environment in the growing medium. Especially the stimulation of *K* may be of importance. Viewing at the hyphal compartment as a root-free substrate microstructure, a root close to those substrate proportions can create a water potential gradient by water uptake. Water would then move along the evolving gradient at higher rates into the vicinity of roots in colonized substrates. Alternatively, a shallower water potential gradient between both substrate proportions is necessary to induce the same water flow. Potential plant water uptake can then increase without the necessity of intrahyphal translocation, which has been estimated to be insignificant (George et al., 1992). This can have important ecological implications, because this would require less investment in root osmotic adjustments to realize the same water flow to roots or effectively enlarges the rhizosphere in colonized substrates with a viable symbiosis. Improvements in hydraulic conductivity via reduced tortuosity or increased connectivity may also appear quicker than hydraulic effects deriving from a hierarchical development of structure, because it may depend less on release of organic material in turnover processes.

Plants sense moisture stress and adjust transpiration via stomatal movement to avoid exhaustive behavior with the resources available (Tardieu and Simonneau, 1998) and mycorrhizal plants often show increased stomatal conductance (Augé et al., 2015). It appears logic, that a substrate with changed water retention and hydraulic conductivity induces a different stress response in the plant. Water contents and hydraulic conductivity are important inputs in plant based stomata (e.g., Tardieu and Simonneau, 1998) and root water uptake models (e.g., van Lier et al., 2006; de Jong van Lier et al., 2008). In addition to direct effects of mycorrhiza on root morphology, root hydraulic conductivity and plant aquaporin expression (e.g., Porcel et al., 2006; Aroca et al., 2007; Bárzana et al., 2012; Ruiz-Lozano et al., 2016) modulating plant water uptake, there may be effects downstream to roots that contribute to the often observed stimulation of plant water, nutrient uptake and drought tolerance in AMF plants. Both, mycorrhizal roots capable of more efficient water uptake and less resistance to water flow in the substrate, may be required for the frequent observation of higher soil drying rates in mycorrhizal systems.

The conservation of water retention and stimulation of unsaturated hydraulic conductivity in AMF symbioses can be a beneficial effect that qualifies AMF as biostimulants in plant production systems. If this also occurs in soils in the field, AMF constitute an effective enlargement of the rhizosphere for water and solute acquisition from the periphery in addition to P. In pot productions systems, this could stimulate water and solute acquisition per unit time, when fertilization and irrigation regimes avoid detrimental degrees of water and nutrient depletion.

## Author Contributions

MB conducted the experiments, analyzed the data, and wrote the manuscript. PF and JG supervised the work, helped developing the experiments, revised the manuscript, and contributed to writing.

## Conflict of Interest Statement

The authors declare that the research was conducted in the absence of any commercial or financial relationships that could be construed as a potential conflict of interest.
